# Non-Destructive X-ray Characterization of a Novel Joining Method Based on Laser-Melting Deposition for AISI 304 Stainless Steel

**DOI:** 10.3390/ma14247796

**Published:** 2021-12-16

**Authors:** Muhammad Arif Mahmood, Diana Chioibasu, Sabin Mihai, Mihai Iovea, Ion N. Mihailescu, Andrei C. Popescu

**Affiliations:** 1 National Institute for Laser, Plasma and Radiation Physics (INFLPR), Magurele, 077125 Ilfov, Romania; arif.mahmood@inflpr.ro (M.A.M.); diana.chioibasu@inflpr.ro (D.C.); sabin.mihai@inflpr.ro (S.M.); ion.mihailescu@inflpr.ro (I.N.M.); 2 Faculty of Industrial Engineering and Robotics, University Politehnica of Bucharest, 060042 Bucharest, Romania; 3 Accent Pro 2000 SRL, Nerva Traian Nr. 01 K6, Ap. 26, Sector 3, 031041 Bucharest, Romania; mihai.iovea@accent.ro

**Keywords:** laser welding, laser-melting deposition, AISI 304 stainless steel sheets, process optimization, X-ray imaging characterization

## Abstract

In this study, an application of the laser-melting deposition additive manufacturing technique as a welding method has been studied for the laser welding (LW) of AISI 304 stainless steel, specifically 0.4 mm and 0.5 mm thick sheets. The welding was carried out without and with filler material. Inconel 718 powder particles were used as filler material in the second case. A series of experiments were designed by changing the process parameters to identify the effect of operating conditions on the weld width, depth, and height. The welds were examined through metallographic experiments performed at various cross-sections to identify the defects and pores. All the deposited welds were passed through a customized mini-focus X-ray system to analyze the weld uniformities. The optimal operating conditions were determined for 0.4 mm and 0.5 mm sheets for the LW with and without filler material. It was found that laser power, laser scanning speed, powder flow rate, and helium to argon gases mixture-control the weld bead dimensions and quality. X-ray analyses showed that the optimal operating conditions gave the least peak value of non-uniformity in the laser welds. This study opens a new window for laser welding via additive manufacturing with X-ray monitoring.

## 1. Introduction

Laser welding (LW) combines metallic or thermoplastic pieces via a laser beam [1]. The laser beam delivers the focused heat, thus allowing for narrow and deep welds. The LW success majorly relies on careful process parameter consideration [2]. A set of optimum laser processing parameters can result in laser welds without pore and crack formation [3]. The LW of any material requires a complete understanding of its thermal, mechanical, and physical properties [4].

The operating parameters, especially the speed of the temperature increment, affect the weld bead dimensions, gas flow rate, welding velocity, specimen hardness, and microstructure formation. To this purpose, various studies have been carried out. The laser welding of AISI 304 stainless steel 1.6 mm thick sheets were carried out using the continuous wave (CW) solid-state Nd: YAG laser [5]. The effect of the laser power and welding speed was studied on weld depth and width. A direct correlation was found between laser power, weld depth, and width. However, welding speed showed an inverse relation with weld depth and width. These two operating conditions were identified as the parameters controlling the weld quality and efficiency. The LW process’s heat-affected zone (HAZ) size was identified based on the laser scanning speed and laser power [6]. The HAZ maximum width and depth decreased by 32% and 62%, respectively, when the laser scanning speed increased from 10 to 100 mm/min by keeping the laser power constant (=10 W). There is no residual stress formation in the HAZ if the critical temperature is less than 840–890 °C. A study reported texture formation in aluminum alloys (AA5182-O and AA6111-T4) welded using the Nd: YAG laser source [7]. An electron backscattering diffraction (EBSD) technique was used to assess the texture. The measurements were carried out as a function of sample thickness. The results presented that the laser welds can develop a substantial texture, particularly the columnar grains entering the base plate into the laser weld. Additionally, they had a 001 texture along with the growth direction. The LW of aluminum-lithium sheets was carried out with a 3 kW CO_2_ laser beam [8]. The influence of various gases was evaluated on the weld appearance, fusion regime dimensions, solute vaporization, hardness, tensile characteristics, and pore formation. It was found that the energy density input plays a direct role in defining the characteristics mentioned above. The effect of laser pulse frequency, laser input energy, and scanning speed was examined on low carbon steels’ mechanical and metallurgical properties [9]. A series of experiments were designed using the Taguchi technique. It was determined that the effective pulse energy is a governing factor in determining the laser-welded joints’ strength. AISI 304 stainless steel sheets laser welding was carried out using a CW CO_2_ laser [10]. They determined that the absorbance of CO_2_ in conduction and keyhole welding was 15% and 65%, respectively. Furthermore, the steel surface pre-oxidation resulted in 30–50% better CO_2_ absorbance.

One of the best ways to analyze the weld quality, using non-destructive techniques, is to combine the welding process with X-ray analyses. In this regard, a high-speed X-ray imaging system was used to observe the keyhole variabilities concerning the defects formation during the LW of copper sheets [11]. Breakthrough results were presented, explaining the bubble generation at the capillary tip. LW penetration as a function of welding speed was studied [12]. X-ray imaging was compiled with the welding process to study the melt pool surface and ejected plume behavior. A wide range of laser scanning speeds was utilized (0–50 m/min) for observation. An inverse correlation was found between laser scanning speed and penetration depth for higher laser scanning speeds. In contrast, when the welding speed is decreased, the weld pool dynamics and plume interaction disturb the keyhole generation stability. At this point, the weld penetration depth showed a random behavior, thus breaking the prior-defined trend. The effect of laser beam pulse shape was analyzed on the keyhole formation via an in situ micro-focused X-ray system [13]. The AISI 304 stainless steel sheet experimentation was irradiated using the Nd: YAG laser beam (pulse duration = 1.1 ms and 4.6 kW peak power = 4.6 kW). It was determined that the keyhole generation started at 0.6–0.7 ms, became deep at 1.5 ms, and finally collapsed at 1 ms. It was also analyzed that the pores were formed at the beam profile, where the peak laser power declined rapidly. The laser welding and weldability phenomena were interpreted concerning the association of physical and metallurgical points of view [14]. Additionally, a micro-focused X-ray in situ monitoring system was used to observe keyhole, melt-flow, bubbles, and pore formation. It was determined that the input parameters, such as laser power, scanning speed, and gas flow rate, play a key role in defining the stability of keyhole, melt-flow, and bubbles and pores generation. The dynamics of keyhole generation and melt pool formation in the LW process was studied by combining the X-ray imaging system with LW [15]. Additionally, the real cause of pores formation was also studied. It was found that during the LW process, a laser hole (keyhole) is generated in the melt pool due to an intense evaporation recoil pressure. This keyhole formation leads to a penetration depth with a high aspect ratio. This characteristic is the most beneficial feature of high-energy-density laser beams (HEDLB). The keyhole in the liquid is unstable, causing cavities or pores in the solidified weld, which is a severe problem of HEDLB.

Errico et al. [16] carried out the LW of AISI 304 stainless steel with AISI 316 L filler powder. To achieve defect-free weld seams, the influence of process parameters, including laser power, laser scanning speed, powder feeding rate, gas flow rate, and laser beam spot size, was investigated on the geometry, microstructure, and porosity inside the weld seams. It was identified that the change in the laser beam spot size is critical in producing various welding regimes. The profile of the laser keyhole is responsible for generating the porosity in the welded region. Prabakaran and Kannan [17] used the Taguchi-based grey relational analysis to optimize the laser welding process parameters for austenitic stainless steel (AISI316) and low carbon steel (AISI1018) materials. Butt joint testing was performed after utilizing a 3.5 kW diffusion-cooled slab CO2 laser and the laser power, welding speed, and focal distance were varied. The authors determined that the ideal operating conditions are laser power = 2600 W, laser scanning speed = 1.5 m/min, and focal distance = 20 mm with the tensile strength = 475.112 MPa. Landowski [18] presented the results for the microstructure of laser beam-welded stainless steel under various welding conditions. Welded joints were produced using an Ytterbium fiber laser without using filler material. The test material was 2205 ferritic-austenitic duplex stainless-steel plates with an 8 mm thickness. It was discovered that the optimum focusing position is one of the most important parameters for shaping high-quality welds. A focus position above the specimen surface (+3, +6 mm) is preferred for high-power fiber laser welding of thick plates. Ding et al. [19] performed the laser welding of heterogeneous metals. Different thermal gradients were attained by varying the operating conditions, including laser welding speed, frequency, and pulse width. The results showed that the melt pool was asymmetric due to the melting of the brass alloy. The measured temperature and melt volume were higher due to the brass alloy’s lower melting temperature and high heat transfer rate. Intermetallic complexes were also present in the melt pool microstructure. The melt pool dimensions can be increased by increasing the peak power and decreasing the welding speed.

This study presents a novel technique for AISI 304 stainless steel, with 0.4 mm and 0.5 mm thick sheets and laser welding. The laser melting deposition (LMD) technique, an additive manufacturing method, has been implemented for sheets’ LW. The welding was carried out without and with filler material. For the second category, Inconel 718 powder particles were added coaxially. In the case of LW without filler, the laser scanning speed, argon gas flow rate, and robot axis angle were varied, while laser power was kept fixed. In contrast, regarding LW with filler, the laser power, laser scanning speed, powder flow rate, and helium and argon gases mixture were changed. A series of experiments were designed by changing the operating conditions to identify their effect on the weld width, depth, and height (with filler material only). The metallographic experiments were conducted at various sections for the deposited welds to analyze the defects and pores in the cross-section. All the deposited welds were subjected to the customized mini-focus X-ray system to analyze the weld uniformities. Last but not least, a set of optimal parameters was produced in the case of LW without and with filler material based on the information generated via experimental analyses.

## 2. Materials and Methods

This study focused on determining the optimal laser welding parameters for AISI 304 stainless steel (SS) sheets’ butt-welding. These sheets were provided by NIKO AUTO COM S.R.L., a metal parts producer in Romania, with the following dimensions: length = 100 mm, width = 50 mm, and thickness = 0.4 mm and 0.5 mm. The chemical composition of AISI 304 SS sheets is presented in Table 1.

Laser welding was executed using a Yb: YAG laser source (TruDisk 3001, Trumpf, Ditzingen, Germany) emitting in continuous mode with wavelength λ = 1030 nm, integrated with a KUKA robot and a 3-beam powder nozzle. The laser beam’s focused spot was 800 µm with a top-hat shape. The deposition optics in the LMD system were mounted on the robotic arm integrated and expressing 6 degrees of freedom through an electromagnetic plate. The laser beam was guided and focused on the workpiece through a system of lenses and mirrors, and the powder stream was blown into the laser focal spot via a 3-beam nozzle. Helium and argon carrier gases transported the metallic powder. The welding experiments were carried out with and without the powder addition by varying the laser power, scanning speed, laser focal point, and carrier gases. Inconel 718 powder particles in the range of 45–90 µm were used as a filler material. The chemical composition of Inconel 718 is provided in Table 2.

**Figure 1:**
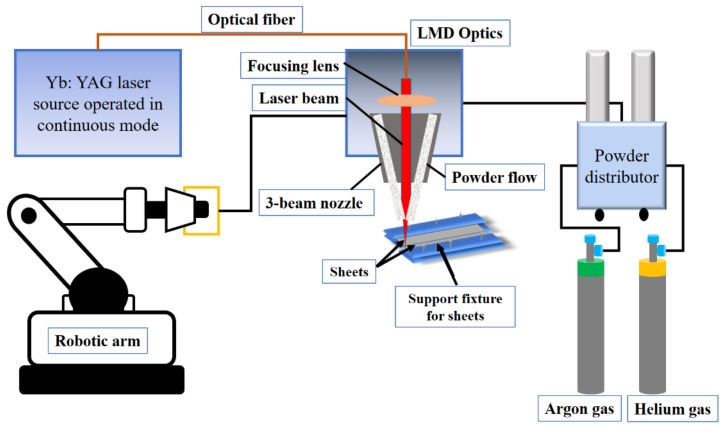
Laser welding via LMD set-up.

Before carrying out the welding experiments, all the sheets were cleaned with ethyl alcohol to eliminate oil and grease particles. Then, these samples were fixed in position using support fixtures. The experimental assembly used for the welding is shown in Figure 1.

Table 3 presents the list of parameters used for the laser welding optimization without the powder addition. The main objective is to obtain fully penetrated welds without defects. During the laser welding, priority was given to (a) obtaining continuous and flawless weld seams and (b) streamlining the welding process in terms of processing time, energy consumption, and total costs. For this purpose, in the case of 0.5 mm thick sheets, the laser power was kept constant (=1000 W) while the laser scanning speed (P1-P4), robot axis angle (P5–P8), and gas flow rate (P9–P12) were varied to determine the optimized set of parameters. The optimized set of parameters was used as the starting point for 0.4 mm thick sheets and only the laser scanning speed was changed to speed up the welding process (W1 and W2).

Table 4 shows the process parameters in stainless-steel samples’ laser welding with a filler material (Inconel 718). The filler metal powder was transported from the powder distributor via a three-jet powder nozzle using a mixture of helium and argon gases. In this case, the laser power, scanning speed, powder flow, and carrier gas flow rates were varied. Initially, the process optimization was conducted for a 0.4 mm thick sheet. The optimized parameters, attained from the welding of 0.4 mm sheets, were used for 0.5 mm sheet welding, except the laser scanning speed was diminished progressively to achieve a better beam penetration into the material.

**Figure 2:**
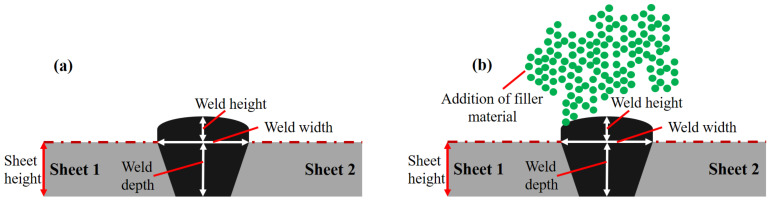
Schematic and nomenclature of weld cross-section: (**a**) without filler and (**b**) with filler material.

Figure 2a,b show the schematic and nomenclature of a typical laser weld in the case of with and without filler material, and can be divided into three sections: (a) weld height, (b) weld width, and (c) weld depth. The effect of primary operating conditions on these three features was studied.

**Figure 3:**
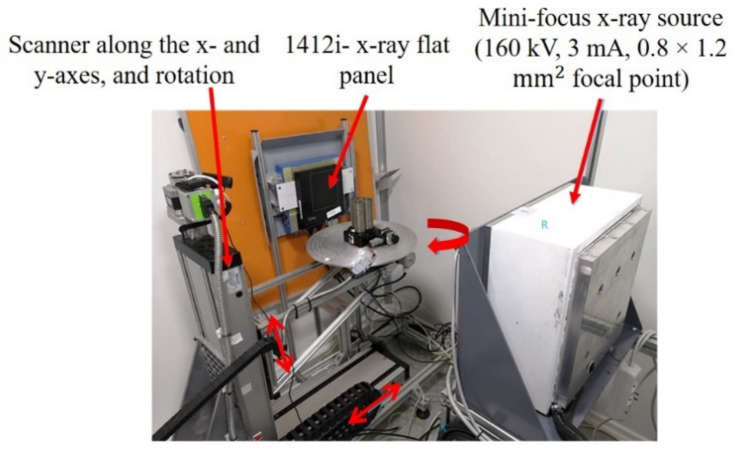
Mini-focus X-ray equipment for radiographic analyses.

After performing the laser-welding experiments, the welds were subjected to radiographic analyses via customized mini-focus X-ray equipment developed by ACCENT PRO, Romania, as shown in Figure 3.

Table 5 presents the X-ray setup description applied to conduct the X-ray experimentation.

The samples images are given in attenuation values (*Att*) computed for each detector’s pixel by using the following formula:

(1) $$Att=ln\left[\frac{I_{o}-I_{off}}{I_{s}-I_{off}}\right],$$

where *I_o_* is the image taken with X-ray source ON and without any object/sample in the beam, *I_s_* is the image taken with X-ray source ON and with the sample placed in the beam, and *I_off_* is the image taken with X-ray source OFF.

**Figure 4:**
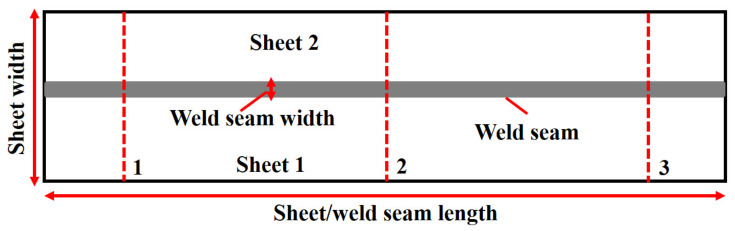
X-ray analyses’ schematic on the weld seam at various locations: (1) at the beginning, (2) in the center, and (3) at the end of the sheet.

X-ray analysis was performed on the whole sample to analyze the weld quality. However, three different sections, namely the (1) start, (2) middle, and (3) end of the weld seam, were chosen to calculate the weld uniformity, as presented in Figure 4. These analyses were carried out orthogonal to the weld seam, resulting in weld seam discontinuities. X-rays are electromagnetic beams and work on electromagnetic radiation’s principles [22]. Electromagnetic radiation, similar to radio waves, visible light, or microwaves, uses waves and photons to carry energy through space, also known as radiant energy. When the interaction between X-rays and a given material occurs, they lose a certain amount of energy based on the material’s absorption behavior. Besides various other parameters, this absorption coefficient depends on the surface uniformity/regularities in such a way that a uniform surface yields a higher absorption coefficient value [23]. X-ray absorption, in general, quantifies the loss of energy at the incident region. Hence, the attained image contrast can assist in quantifying the weld quality.

## 3. Results and Discussion

**Figure 5:**
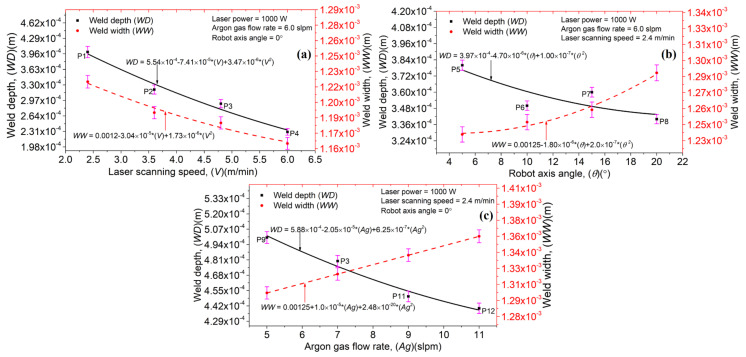
Laser welds’ depth and width by changing: (**a**) laser scanning speed, (**b**) robot axis angle, and (**c**) argon gas flow rate.

Figure 5a–c exhibit the influence of the laser scanning speed (*V*), robot axis angle (θ), and argon gas flow rate (*Ag*) on the laser welds’ depth (*WD*) and width (*WW*). It should be noted that laser power (*P*) was kept constant. For a given *P*, *Ag,* and θ, an increment in *V* causes a declination in the *WD* and *WW* values. The *V* plays a vital role in defining the laser–material interaction time. A higher *V* defines less laser–material interaction time, resulting in a melt pool formation with smaller dimensions. In Figure 5a, a curve fitting was performed to analyze the comprehensive and deterministic outcomes, resulting in a polynomial expression that can predict the different outputs based on given inputs. Figure 5b shows a correlation between θ, *WD,* and *WW*. “θ” is defined as the inclination angle provided by the robot axis. In the current study, θ did not show any significant impact over *WD* and *WW* dimensions. Additionally, a random behavior was observed in the datasets. A curve fitting was performed to identify the trend in the generated data points. An increase in θ causes a decrease in *WD* and an increase in *WW*, keeping the *P*, *Ag,* and *V* constant. When the robot arm is perpendicular to the metallic sheets welded, the laser spot focuses on a particular circular area. However, with an increment in the robot arm axis angle, the laser spot no longer remains circular; instead, an elliptical shape is attained, resulting in higher *WW* and lesser *WD* than the last case. Furthermore, *Ag* presented an inverse relation with *WD* and direct relation with *WW* for a given *P*, *V,* and θ, as shown in Figure 5c. It can be attributed to the increase in argon content in the weld pool and the formation of a thick protective layer on the weld surface, reducing the *WD*. These findings are in good correlation with the one provided in Reference [24]. The data trend can be analyzed via a curve fitting, as shown in Figure 5. Furthermore, error bars have been plotted in Figure 5 that show the standard deviation in the data attained by varying the operating conditions. In other words, the short error bars demonstrate that the concentration of the values is high compared to the long error bars.

**Figure 6:**
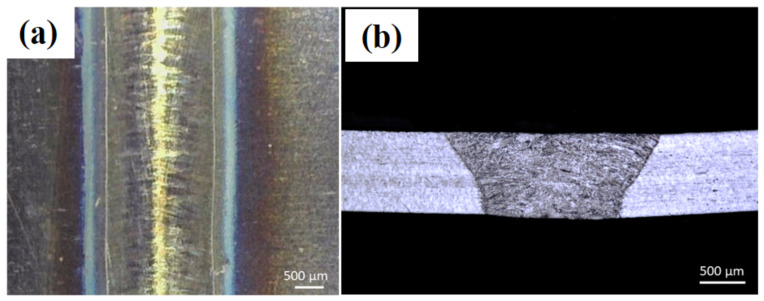
Optical images of laser-welded 0.5 mm thick sheets’ cross-sections without powder addition and with laser power = 1000 W, laser scanning speed = 2.4 m/min, gas flow rate = 5 slpm, and robot tilt angle = 0°: (**a**) top view and (**b**) cross-section.

Using the operating conditions defined in Table 3, several laser welds combined with metallography experiments were carried out. The optimal parameters for laser welding of AISI 304 stainless steel sheets without filler material, with a thickness of 0.5 mm, are: (a) laser power = 1000 W, (b) laser scanning speed = 2.4 m/min, (c) argon gas flow rate = 5 slpm, and (d) robot tilt angle = 0°. Figure 6a,b show the optical images of the top view and cross-section of the welded sheets by applying optimal laser processing parameters. It can be observed that the weld is uniform, without pores and cracks. When the V increases beyond 3 m/min, the laser–material interaction time decreases, ultimately reducing the laser energy density transfer into the material, thus leading to narrower weld seams which can lead to incomplete penetration across the sheet thickness. When the laser energy density cannot travel across the sheet thickness, it results in a lack of fusion between the two provided sheets, thus giving incomplete laser weld penetration that can be analyzed near the bottom end of the sheets with reference to the laser–material interaction surface.

**Figure 7:**
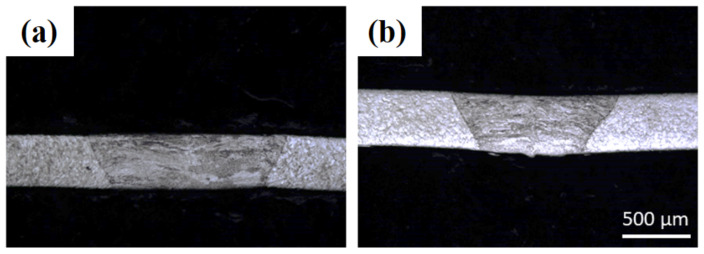
Optical images of 0.4 mm thick sheets’ laser weld cross-sections without powder addition and with laser power = 1000 W, gas flow rate = 5 slpm, robot tilt angle = 0° and (**a**) laser scanning speed = 2.4 m/min and (**b**) laser scanning speed = 3.0 m/min.

To determine the laser welding operating conditions for AISI 304 stainless-steel sheets with 0.4 mm thickness, the optimal conditions identified for the 0.5 mm thickness sheets were initially used, as displayed in Figure 7a. As the sheet thickness (=0.4 mm) is smaller than 0.5 mm, the laser scanning speed was increased from 2.4 to 3.0 m/min, as presented in Figure 7b. It can be observed that the weld width decreases with the increase in scanning speed, indicating a complete and flawless welding penetration. However, beyond 3.0 m/min, an incomplete laser depth was achieved. Hence, the optimal parameters for the laser welding of AISI 304 stainless steel sheets without filler material, with a thickness of 0.4 mm, are: (a) laser power = 1000 W, (b) laser scanning speed = 3.0 m/min, (c) argon gas flow rate = 5 slpm, and (d) robot tilt angle = 0°.

**Figure 8:**
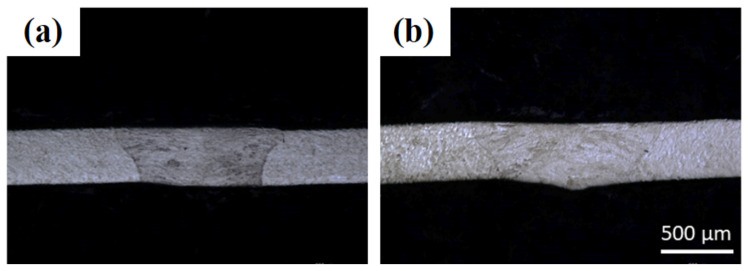
Optical images of 0.4 mm thick sheets’ laser weld cross-sections without powder addition and with laser power = 1000 W, gas flow rate = 5 slpm, robot tilt angle = 0°, and laser scanning speed = 3.0 m/min: (**a**) focal plane + 5 mm and (**b**) focal plane + 10 mm.

For weld joints, good positioning of the sheets is essential to obtain completely penetrated welds. A larger spot size results in easier sheet positioning for laser welding. Therefore, the laser beam was defocused to increase the spot diameter. In LMD equipment, laser defocusing decreases the energy density at the sheet surface. Figure 8a,b show the results of a defocused laser beam with (focal plane + 5 mm) and (focal plane + 10 mm) distances, respectively. The spot diameter was increased from 0.8 to 1 mm (focal plane + 5 mm) and 1.15 mm (focal plane + 10 mm). Acceptable weld seams were achieved via a defocused laser beam, thus relaxing the exact positioning of the laser spot at the two plates’ junction. However, this strategy can only be implemented using a “top-hat” laser spot in which the laser intensity is uniformly distributed. Extending this technique to other distributions (Gaussian or various non-uniformities) is difficult and often impossible.

**Figure 9:**
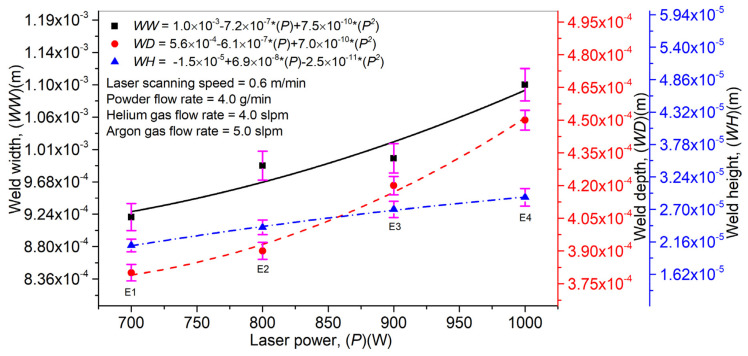
Effect of laser power on laser weld width, depth, and height in laser welding with filler material (Inconel 718).

After determining the optimal laser welding parameters without filler materials, experiments were performed to determine the operating conditions with filler material. To this purpose, a mixture of helium and argon gases was used to transport powder particles from the powder feeder to the AISI 304 stainless steel sheets to be welded. Initially, the optimization was carried out for 0.4 mm thick sheets. Figure 9 explains the effect of the laser power (*P*) on the weld width (*WW*), depth (*WD*), and height (*WH*). A direct relation can be observed between *P*, *WW*, *WD*, and *WH* by keeping the laser scanning speed (*V*), powder flow rate (*Pf*), and a mixture of helium (*Hg*) and argon (*Ag*) gases constant. With a rise in *P*, the energy density for a given area also increases, forming a melt pool with higher width and depth. The LMD technique includes the coaxial inclusion of powder particles which pass across the laser beam, resulting in laser beam attenuation. The laser beam is accountable for particles’ melting, thus causing weld formation. However, not all the powder particles passing across the laser beam contribute to weld generation and give powder utilization efficiency, defined as the ratio between the total powder provided from the powder feeder to the powder used in layer formation. With an increase in P, the laser energy density rises and causes an improved powder particle melting. It, in return, increases the weld height. Error bars have been provided in Figure 9 to identify the effect of *P* on *WW*, *WD*, and *WH*. The short error bars show that the change in *P* is not significantly affected and vice versa for long error bars.

**Figure 10:**
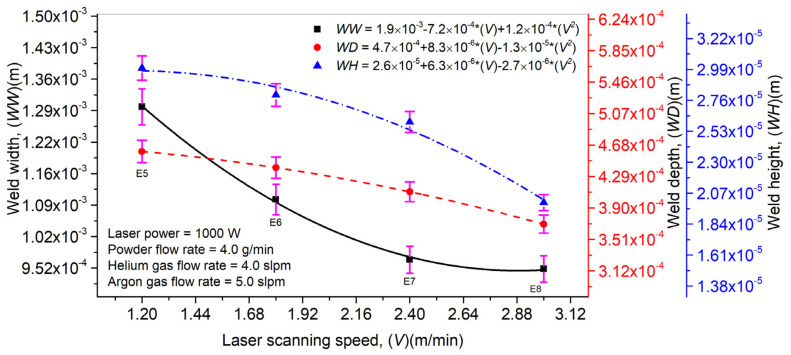
Effect of laser scanning speed on laser weld width, depth, and height in laser welding with filler material (Inconel 718).

The influence of *V* on *WW*, *WD,* and *WH* is highlighted in Figure 10. Additionally, *P*, *Pf*, and a mixture of *Hg* and *Ag* were kept constant. It can be observed that an increment in *V* decreases the laser–material interaction time, which in turn reduces the melt pool dimensions and powder particles’ melting, thus diminishing the *WW*, *WD,* and *WH* values. An optimum *V* value is necessary to determine as a prolonged laser–material time usually causes residual stress formation in the weld and disturbs the welded part’s mechanical properties. Figure 10 shows the error bars plotted to analyze the effect of *V* on *WW*, *WD*, and *WH*. It can be seen that an increase in *V* affects *WW* significantly compared to the *WD* and *WH*.

**Figure 11:**
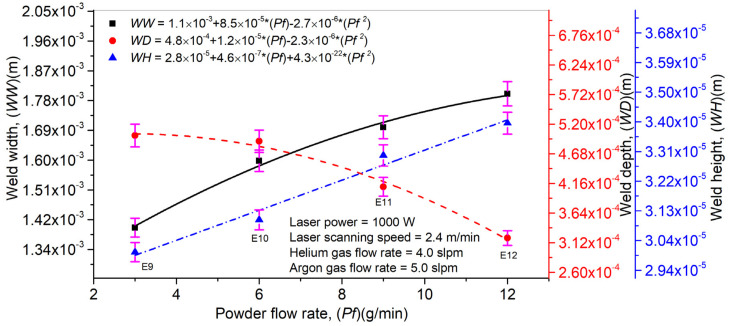
Effect of powder flow rate on laser weld width, depth, and height in laser welding with filler material (Inconel 718).

An influence of *Pf* on *WW*, *WD,* and *WH* is plotted in Figure 11. A direct correlation can be analyzed between *Pf*, *WW,* and *WH*. In contrast, *Pf* presented an inverse relationship with *WD*. As explained above, laser beam attenuation is an inherited phenomenon in the LMD technique, which prevails in the deposition process with the increase in *Pf*. Excess laser beam energy reaches the substrate’s surface and does not find enough time to arrive at the depth. It causes an elevation in *WW* and *WH* while *WD* declines steadily. Error bars have been drawn in Figure 11 to illustrate the influence of *Pf* on *WW*, *WD*, and *WH*. A short error bar means that the concentration of values is high and the average value is more certain, while it is the opposite for long error bars.

**Figure 12:**
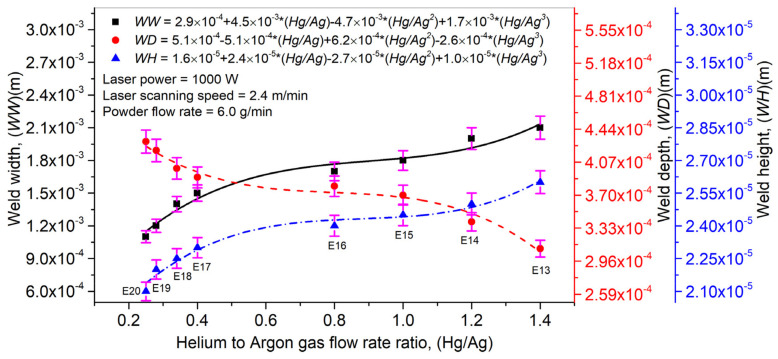
Effect of helium to argon gas flow rate ratio on laser weld width, depth, and height in laser welding with filler material (Inconel 718).

Figure 12 displays a link between the *Hg*/*Ag* ratio on *WW*, *WD,* and *WH*. For a given set of *P*, *V,* and *Pf*, the *Hg*/*Ag* ratio showed a direct link with *WW* and *WH*, while an inverse link was presented between the *Hg*/*Ag* ratio and *WD*. With an increase in the gas mixture ratio, a thick protective layer is formed at the melt pool surface, not allowing the laser beam energy to travel in the substrate’s depth. It causes the entire powder deposition at the substrate’s surface. It is the possible explanation behind the behaviors presented by *WW*, *WD,* and *WH*. In Figure 12, error bars have been presented to display the effect of the *Hg*/*Ag* ratio on *WW*, *WD,* and *WH* using standard deviation. It can be analyzed that the *Hg*/*Ag* ratio affects *WD* significantly compared to *WH* and *WW* quantities.

**Figure 13:**
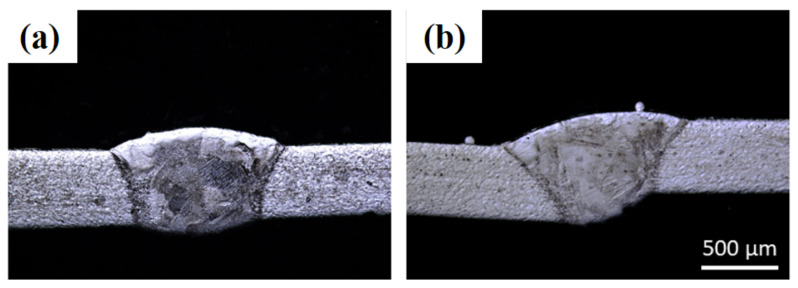
Optical images of 0.4 mm welded plates using Inconel 718 filler material with laser power = 1000 W, laser scanning speed = 2.4 m/min, powder flow rate = 6 g/min, helium gas flow rate = 4 slpm, argon gas flow rate = 10 slpm, and robot tilt angle = 0°: (**a**) perfect positioning and (**b**) positioning with a height difference of ~100 µm.

After carrying out various experiments with metallography analysis, the following optimal laser processing parameters for the 0.4 mm thick sheet were determined: (a) laser power = 1000 W, (b) laser scanning speed = 2.4 m/min, (c) powder flow rate = 6 g/min, (d) helium gas flow rate = 4 slpm, (e) argon gas flow rate = 10 slpm, and (f) robot tilt angle = 0°. The optical imaging and metallography analysis are shown in Figure 13a,b. To identify the difference between the weld seam without filler material, the results in Figure 13 can be compared with those provided in Figure 7. In Figure 13a, both sheets are positioned precisely. However, in Figure 13b, both sheets are placed in perfect position and adjusted at a difference of 100 µm with respect to height. Hence, one can conclude that using the filler material can provide a reasonable solution to the difficulties that arise from improper positioning in the case of laser welding without filler material. The same operating conditions as for 0.4 mm thick sheets were implemented for 0.5 mm thick sheets, except the laser scanning speed was varied by (i) 1.2 m/min and (ii) 2.4 m/min. However, a thorough and defect-free weld was achieved with a 2.4 m/min scanning speed. Hence, the optical parameters were kept the same as for the 0.4 mm thick sheet.

**Figure 14:**
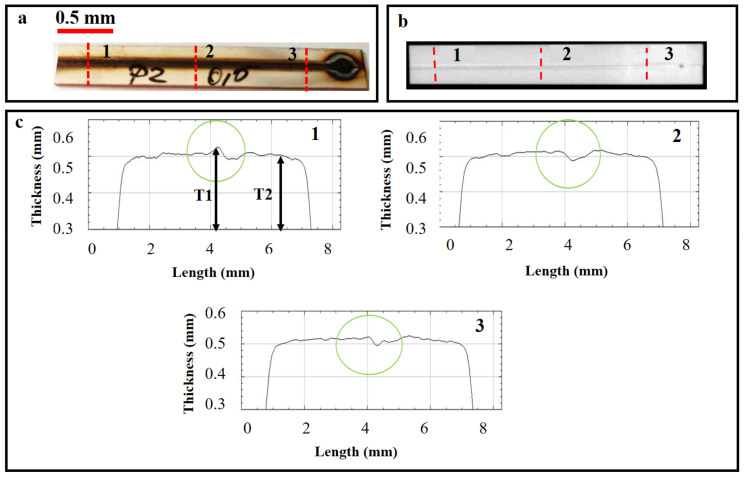
P2 sample (**a**) laser weld by the LMD, (**b**) X-ray analysis of weld using mini-focus X-ray equipment, and (**c**) graphical results of various scans at different positions (1, 2, and 3).

To analyze the effect of weld quality, the seams were subjected to X-ray analyses. However, only the P2 sample is shown here due to space limitations. Figure 14a,b show the image of a laser weld using the LMD equipment and the corresponding radiographic image, respectively. The weld uniformities were plotted between the scan length and thickness (weld) at three different locations (1, 2, and 3), as shown in Figure 14c. These surface uniformities explain the irregularities within the weld seam based on the X-ray absorption coefficient at the incident regime so that a uniform surface yields a higher absorption coefficient value [23]. Hence, the X-rays can be utilized to quantify the weld seams. It is worth mentioning that X-rays present several advantages over a traditional profilometer setup. One of the advantages is that X-rays can be applied for in situ measurement and characterization of the weld seam. In addition, X-rays can pass across the weld seams, just providing analysis across the samples. As highlighted with green circles, these weld uniformities were used to identify the weld quality. Furthermore, from the graphs, the peak (*T*1) and valley values were calculated to compute the peak value of non-uniformity in the weld seams (percentage), as given in Equation (2):

(2) $$Peak\;value\;of\;non-uniformity\;in\;the\;weld\;\left(\%\right)=\left(\frac{T1-T2}{T1}\right)\times 100$$

**Figure 15:**
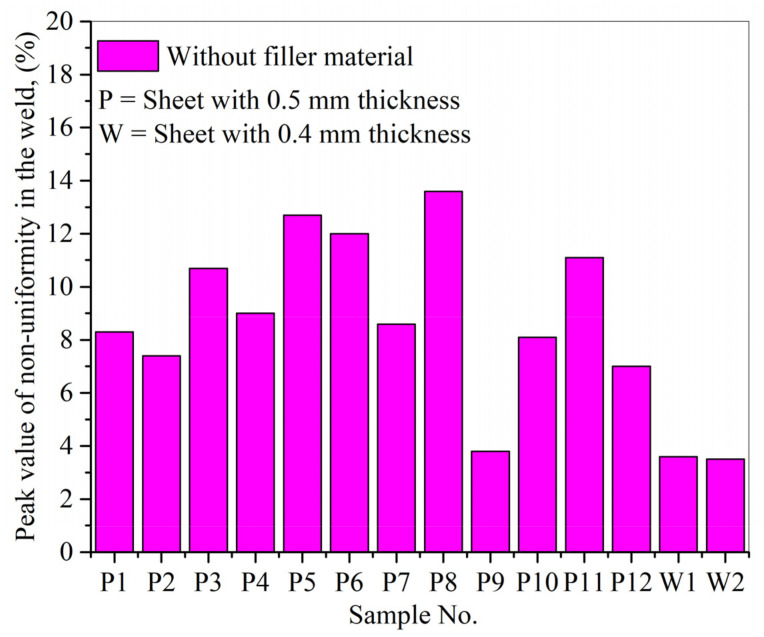
The peak value of non-uniformity was calculated via X-ray analyses for laser welds without filler material.

After utilizing Equation (1), the peak value of non-uniformity in the weld seams (percentage) without filler material was calculated for different locations (1, 2, and 3). An average was calculated, as presented in Figure 15. The optimal weld parameters, given by sample P9 for 0.5 mm thick sheets, gave the least non-uniformity value (=3.8%). Similarly, in the case of 0.4 mm sheets, the optimal parameters, shown by the W2 sample, presented the least non-uniformity value (=3.5%).

**Figure 16:**
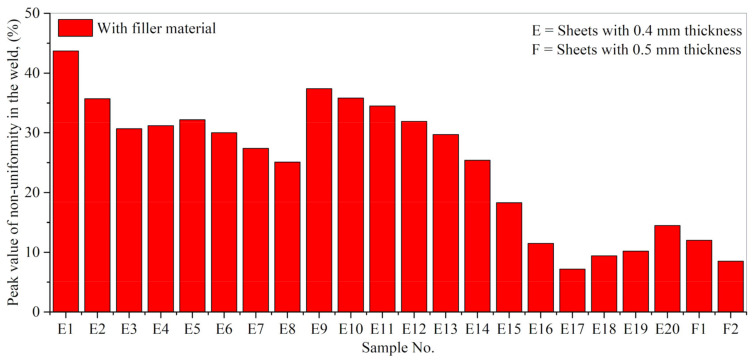
The peak value of non-uniformity was calculated via X-ray analyses for laser welds with filler material (Inconel 718).

Figure 16 collects the peak value of non-uniformity in the welds (percentage) in the case of laser welding with filler material (Inconel 718). In the case of E17 (0.4 mm thick sheets) and F2 (0.5 mm thick sheets), the optimal weld parameters presented the least non-uniformity values equal to 7.3% and 8.5%, respectively. From the above results, one can conclude that X-ray analysis is an efficient non-destructive technique for characterizing weld seams.

Practically, the weld non-uniformity can be defined in terms of the weld quality, surface morphology characteristics, and melting situation of a given material. Each weld presented a non-uniformity value (percentage); however, it primarily depends on the set of operating conditions. Furthermore, the welds without filler showed fewer non-uniformities than those with filler material. The powder particles (filler) are micro-grains fed simultaneously along with the laser beam responsible for particles’ melting and melting pool formation. These melted particles define a new layer in correspondence with the base material. Furthermore, the quality of the melted layer is entirely dependent on the melting situation defined by the operating conditions. The two phenomena, namely the (a) inclusion of a new layer and (b) melting situation of the particles, are responsible for giving high non-uniformity (percentage) compared to the welding without material addition. To achieve weld with minimum non-uniformities, it is desirable to determine optimum process parameters. It is worth mentioning that each set of process parameters, even when repeating the same operating condition, will present a value of non-uniformity (percentage) as the deposited material will have a certain morphology and surface characteristics. These values can be lessened but not eliminated.

## 4. Conclusions

This study carried out laser welding of AISI 304 stainless steel, specifically 0.4 mm and 0.5 mm thick sheets, via the laser-melting deposition (LMD) technique. The welding was carried out without and with filler material. Inconel 718 powder particles were used as filling material in the joints welded. Metallographic experiments were conducted to analyze the defects and pores in the cross-sections. These welds were subjected to X-ray analyses to identify the weld quality in weld uniformities. Based on the study, the following conclusions are deduced:

- In laser welding without filler material, an increment in the laser scanning speed causes a decrease in the weld width and depth. An increment in the robot axis angle decreases the weld depth and increases the weld width. Additionally, the argon gas flow rate presented an inverse relation with weld depth and a direct relation with weld width.
- The optimal parameters for the laser welding of AISI 304 stainless steel sheets without filler material, with a thickness of 0.5 mm, are: (a) laser power = 1000 W, (b) laser scanning speed = 2.4 m/min, (c) argon gas flow rate = 5 slpm, and (d) robot tilt angle = 0°. All the parameters are the same for 0.4 mm thick sheets, except the laser scanning speed increases from 2.4 to 3.0 m/min due to inferior thickness compared to the 0.5 mm thick sheet.
- Good positioning of the sheets is necessary to achieve entirely penetrated welds. A larger spot size results in easier sheet positioning for laser welding. The laser beam was defocused with focal plane + 5 mm and focal plane + 10 mm distances. Acceptable welds were achieved through the defocused laser beam, thus comforting the exact positioning of the laser spot at the sheet area to be welded. This strategy can be implemented with a “top-hat” laser beam profile and loses application using a “Gaussian” intensity distribution.
- For laser welding with Inconel 718 filler material, a direct relation was found between laser power and weld width, depth, and height. Increasing the laser scanning speed decreases the laser–material interaction time, thus diminishing the weld width, depth, and height.
- A direct correlation was analyzed between the powder flow rate and weld width and height, while an inverse relationship was presented between the powder flow rate and weld depth. Laser beam attenuation is an inherited phenomenon in the LMD process caused by laser beam energy absorption by the powder particles. These powder particles melt and an excess of material is deposited on the surface, while the remaining laser energy (unabsorbed by the particles) reaching the surface is not sufficient for completely penetrating and melting the material to be welded. The deposition of molten particles on the surface of the joint causes an elevation in the weld width and height while weld depth declines steadily.
- By utilizing filler material, the optimized set of parameters for 0.4 mm and 0.5 mm sheets are: (a) laser power = 1000 W, (b) laser scanning speed = 2.4 m/min, (c) powder flow rate = 6 g/min, (d) helium gas flow rate = 4 slpm, (e) argon gas flow rate = 10 slpm, and (f) robot tilt angle = 0°.
- X-ray analyses presented a quantitative value for the peak value of non-uniformity in laser welds. In welding without filler material, the optimal weld parameters, given by sample P9 for 0.5 mm thick sheets, gave the least non-uniformity value (=3.8%). Similarly, for 0.4 mm sheets, the optimal parameters, shown by the W2 sample, presented the least non-uniformity value (=3.5%). Furthermore, the optimal weld parameters for welding with filler material in the case of E17 (0.4 mm thick sheets) and F2 (0.5 mm thick sheets) presented the least non-uniformity values equal to 7.3% and 8.5%, respectively.

## Figures and Tables

**Table 1:** Chemical composition of AISI 304 stainless steel sheets [20].

Elements	(%)
Carbon	0.08
Manganese	2.0
Silicon	0.75
Phosphorus	0.045
Sulfur	0.03
Chromium	18.0–20.0
Nickel	8.0–10.5
Iron	Balance

**Table 2:** Chemical composition of Inconel 718 powder particles [21].

Elements	(%)
Carbon	0.08
Manganese	0.35
Phosphorus	0.015
Sulfur	0.015
Silicon	0.35
Chromium	17–21
Nickel	50–55
Molybdenum	2.80–3.30
Columbium	4.75–5.50
Titanium	0.65–1.15
Aluminum	0.20–0.80
Cobalt	1.0
Boron	0.006
Copper	0.30
Tantalum	0.05
Iron	Balance

**Table 3:** Laser welding operating parameters without powder addition.

Sr. No	Sheet Thickness (mm)	Laser Power (*P*; W)	Laser Scanning Speed (*V*; m/min)	Argon Gas Flow (*Ag*; slpm)	Robot Axis Angle(θ; °)	Sr. No	Sheet Thickness(mm)	Laser Power(W)	Laser Scanning Speed (m/min)	Argon Gas Flow (slpm)	Robot Axis Angle(θ; °)
P1	0.5	1000	6.0	6.0	0	W1	0.4	1000	2.4	5.0	0
P2			4.8			W2	0.4	1000	3.0	5.0	0
P3			3.6								
P4			2.4			**⸪** P(Sr. No) = sheets with 0.5 mm thickness; W(Sr. No) = sheets with 0.4 mm thickness. **⸪** number of scanning lines = 1.0.					
P5					5						
P6					10						
P7					15						
P8					20						
P9				5.0	0						
P10				7.0							
P11				9.0							
P12				11.0							

**Table 4:** Laser welding operating parameters with filler material (Inconel 718).

Sr. No.	Sheet Thickness (mm)	Laser Power (*P*; W)	Laser Scanning Speed (*V*; m/min)	Powder Flow Rate (*Pf*; g/min)	Helium Gas Flow Rate (*Hg*; slpm)	Argon Gas Flow Rate (*Ag*; slpm)
E1	0.4	700	0.6	4.0	4.0	5.0
E2		800				
E3		900				
E4		1000	0.6			
E5			1.2			
E6			1.8			
E7			2.4			
E8			3.0			
E9			2.4	3.0		
E10				6.0		
E11				9.0		
E12				12.0		
E13				6.0		
E14					5.0	
E15					6.0	
E16					7.0	
E17					4.0	10.0
E18						12.0
E19						14.0
E20						16.0
F1	0.5		1.2			10.0
F2			2.4			
**⸪** E(Sr. No) = sheets with 0.4 mm thickness; F(Sr. No) = sheets with 0.5 mm thickness. **⸪** number of scanning lines = 1.0.						

**Table 5:** X-ray setup description used to perform X-ray analyses.

Item	Value
X-ray source	
Type	Gilardoni Monoblock AION
Peak voltage	160 kVp
Current	3 mA
Power	500 W
Focal spot size	1.2 mm
Cone beam angle	40°
X-ray detector	
Detection Technology	Flat panel type X-Panel 1512
Size	150 mm × 120 mm
Pixel size	100 µ
ADC type	14 bits
Peak voltage	225 kVp
Geometry	
Source-detector distance (SDO)	800 mm
Object-detector distance (ODD)	40 mm
Acquisition parameters	
Peak voltage	150 kVp
Current	2 mA
Integration time per frame	50 ms
Average frames acquired	25 frames

## Data Availability

Not applicable.
